# Retrospective cohort analysis comparing the incidence of deep vein thromboses between peripherally-inserted and long-term skin tunneled venous catheters in hemato-oncology patients

**DOI:** 10.1186/s12959-015-0052-2

**Published:** 2015-06-25

**Authors:** Priya Sriskandarajah, Katharine Webb, David Chisholm, Ravi Raobaikady, Kim Davis, Natalie Pepper, Mark E Ethell, Mike N Potter, Bronwen E Shaw

**Affiliations:** Department of Haem-Oncology, Royal Marsden Hospital, Downs Road, Sutton, SM2 5PT UK; Department of Anesthetics, Royal Marsden Hospital, Downs Road, Sutton, SM2 5PT UK

**Keywords:** Catheter-related deep vein thrombosis, Cohort study, Doppler ultrasonography, Long term skin tunneled catheterisation, Venous thromboembolism

## Abstract

**Background:**

The introduction of central venous catheters has advanced medical care, particularly in hemato-oncology. However these can be associated with an increased thrombotic risk. Previous studies have compared the rate of thrombotic events between peripherally- inserted (PICCs) and long term skin tunneled catheters (LTSTCs) noting fewer complications associated with the latter, though this has rarely translated into clinical practice. The objectives of our study was to compare the cumulative incidence of thrombotic events between peripherally-inserted and long term skin tunneled venous catheters.

**Patients/methods:**

We performed a retrospective, single center cohort analysis of patients with hematological malignancies who had either a PICC or LTSTC line inserted between January 2010 through January 2013. Cumulative incidences of thrombotic events were compared between the two groups, and post-thrombotic complications were also examined.

**Results:**

346 patients had a PICC inserted with cumulative incidence of symptomatic thrombosis of 5.8%, while 237 patients had a LTSTC inserted with a cumulative incidence of 1.7% (p = 0.003). Post-thrombotic complication rates, particularly infection, were higher in the PICC group compared to the LTSTC group (p = 0.597).

**Conclusions:**

Our study showed that the incidence of thrombotic events in hemato-oncology patients was significantly lower in those who had a LTSTC compared to PICC line. As the use of central venous lines increases in hemato-oncology patient care, a randomized trial comparing PICCs and LTSTCs is necessary to address which venous access is most appropriate in this cohort of patients, with minimal risk of morbidity and mortality.

## Background

The introduction of long term central venous catheters has been one of the most significant advances in medical care leading to its widespread use in a broad range of diseases, including infections and malignancies. Unfortunately, the use of these catheters can be associated with complications, most notably the development of a catheter-related deep vein thrombosis (DVT).

The reported incidence of catheter-related thrombosis (CRT) has been highly variable, ranging from 12-60% in various studies [1,2]. However, it has been commonly noted that patients with malignancy are at an increased risk of developing this complication [3,4]. Furthermore, the impact of a thrombotic event can be clinically significant, particularly in hemato-oncology patients, as they require long-term access for the administration of blood products, chemotherapy and stem cell rescue. However, data for catheter-related thromboses in patients with hematological malignancies has been limited [5–7].

In 2010, Tran et al. were one of the first groups to examine the incidence of CRTs in hemato-oncology patients following insertion of either a long term skin tunneled (LTSTC) or peripheral (PICC) central venous catheter [8]. They found that the incidence of thrombotic events was higher in those with a peripheral compared to central line, with the majority of patients managed by initiating anticoagulation. Several studies have supported these findings with reported rates of asymptomatic and symptomatic PICC-related thromboses of up to 50% and 22% respectively [9–12].

Despite these data however this has rarely translated into clinical practice with the majority of UK hospitals electing to use PICCs as the procedure is considered less invasive. This is due to the reported increased incidence (approximately 3%) of serious mechanical complications in patients undergoing central venous catheter (CVC) placement, including arterial puncture (incidence range 3.1-15%) and pneumothorax (incidence range 0.1-3.1%) [13,14].

Nevertheless the risk of catheter-related thrombosis is not insignificant and can be associated with significant patient morbidity and potential mortality [15]. Based on the current literature, it is likely that the incidence of thromboses will be higher in PICCs compared to LTSTCs, but more data is required [16–18]. Accordingly we performed a retrospective cohort study comparing the cumulative incidence of thrombotic events following insertion of either a PICC or LTSTC line in hemato-oncology patients. We also analyzed secondary outcomes including post-thrombotic infection and pulmonary emboli.

## Patients/methods

### Study design

This cohort study and waiver of informed consent was approved by the Royal Marsden Audit Review Committee. Data were obtained by retrospective review of paper and electronic medical records for all hemato-oncology patients admitted to Royal Marsden Hospital who had either a PICC or LTSTC line insertion between 1^st^ January 2010 to 1^st^ January 2013. Only the first line placed during hospitalization was included in this study to avoid error that may be due to a previous catheterization at another site. Symptomatic thrombosis was determined by reviewing the duplex ultrasound reports for every patient who had a PICC or LTSTC inserted. A catheter-related thrombosis was defined as an acute proximal large vein thrombosis in association with the catheter confirmed by duplex ultrasonagraphy.

### Primary outcome

The primary outcome was the occurrence of a symptomatic catheter-related thrombosis.

### Secondary outcomes

The secondary outcomes were the occurrence of post-thrombotic complications, namely Infection, Hemorrhage, Pulmonary Embolism and Line Removal. Similar to previous studies, line-associated infection was defined as identification of the same bacteria cultured from the line as well as from one or more blood cultures not drawn from the line and with no other identifiable source for the bloodstream infection [14].

### Data collected

The data collected in addition to the primary and secondary outcomes included age, gender, underlying hematological malignancy, disease stage and duration of dwell time of the line. Previous studies had identified other risk factors associated with thrombosis including history of venous thromboembolic events, type of chemotherapy received and concomitant medication (including anti-platelet therapy and low-molecular weight heparin) [3,19]. Subsequently we collected these factors for comparison also.

### Statistical analysis

All data was entered into a spreadsheet audit tool. Summary statistics for quantitative data are described as a median with range, while nominal data are expressed as a percentage. Log-rank test was used to compare Kaplan Meier of the 2 groups while Fishers’ exact test was used to compare the complication rates in the groups. Due to the low number of outcomes, multivariate analysis was not performed. A p-value < 0.05 was considered statistically significant.

## Results

### Baseline characteristics

During the study period 583 hemato-oncology patients had a line inserted at Royal Marsden Hospital, of which 346 had a PICC and 237 had a LTSTC. The baseline characteristics for these two groups have been summarized in Table 1. These were generally similar between the two groups, with the majority of our patients being diagnosed with AML and having relapsed/refractory disease. Notably the dwell time of line was significantly longer in the LTSTC group (median 109.4 days) compared to the PICC group (median 64.9 days).

**Table 1:** **Baseline characteristics for all hemato-oncology patients undergoing line insertion**

**Patient characteristics**	**PICC (n = 346)**	**LTSTC (n = 237)**
Median age (range)	49.7 (15-77)	50.8 (17-72)
Gender:		
Females (%)	49	49
Males (%)	51	51
Hematological malignancy:		
AML (%)	36	25
ALL (%)	14	10
HL (%)	5	7
NHL (%)	24	15
MM (%)	15	27
Chronic leukemia (%)	4	9
Misc (%)	2	7
Disease stage:		
Newly diagnosed (%)	27	10
Relapsed/Refractory (%)	52	53
Remission (%)	21	37
Median duration of dwell time of line in days (range)	64.9 (1-519)	109.4 (1-783)

NB: AML = Acute Myeloid Leukemia; ALL = Acute Lymphoblastic Leukemia; HL = Hodgkin Lymphoma; NHL = Non-Hodgkin Lymphoma; MM = Multiple Myeloma; Misc. = Miscellaneous consisting of Myelofibrosis, Myelodysplastic Syndrome and Aplastic Anemia; Chronic Leukemia consisting of Chronic Myelomonocytic Leukemia, Chronic Myeloid Leukemia, Chronic Lymphocytic Leukemia and T-Prolymphocytic Leukemia; PICC = Peripherally-Inserted Central Venous Catheter; LTSTC = Long-term skin-tunneled venous catheter.

### Primary outcome

**Figure 1:**
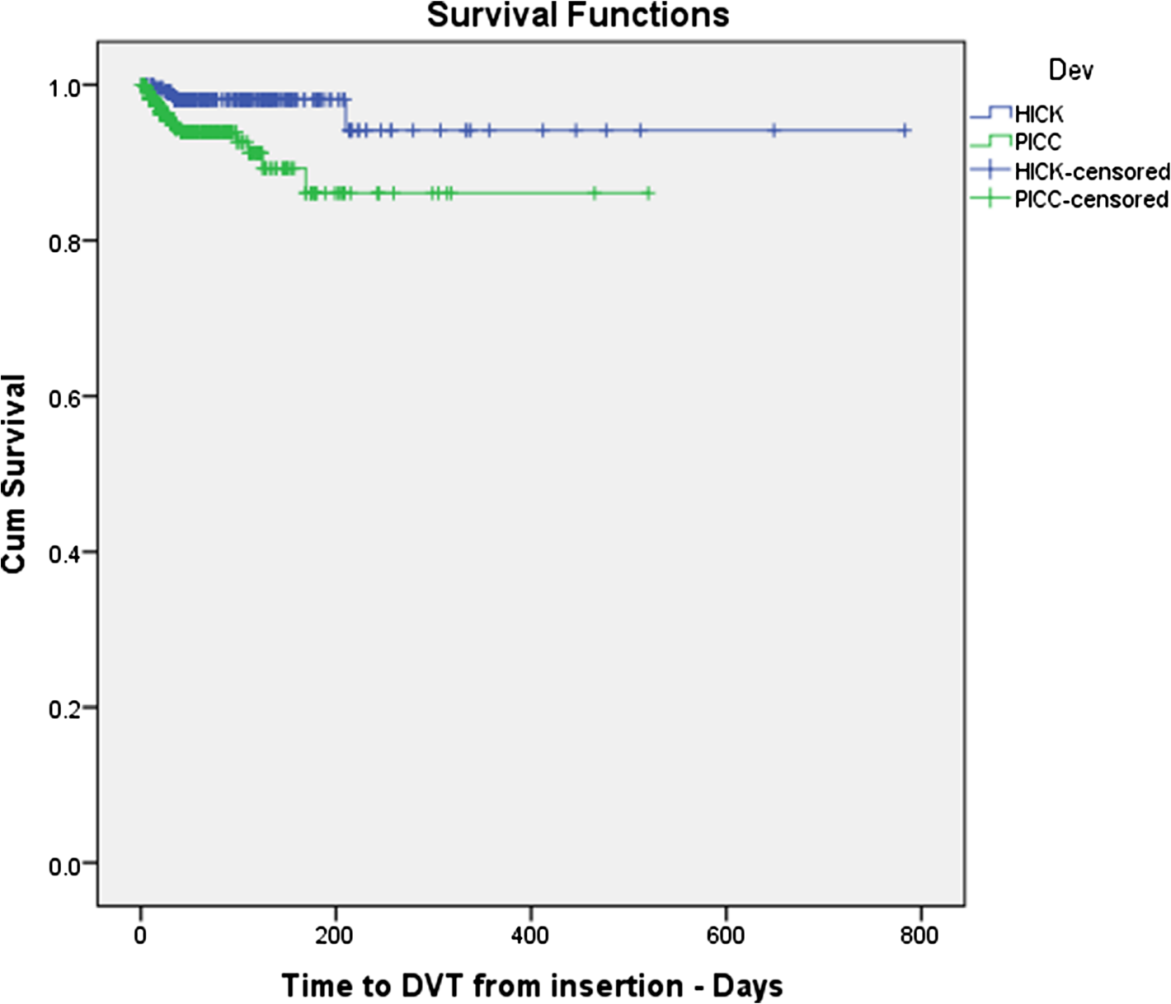
Kaplan Meier Plots comparing the cumulative incidence of DVT events between patients who had either a PICC or LTSTC (e.g. Hickman) line inserted. A total 583 patients were identified within the reviewing period, of which 346 had PICC and 237 had LTSTC. Log rank test p = 0.003. NB: PICC = peripherally-inserted central venous catheter; LTSTC = long term skin tunneled catheter (i.e. Hickman line).

In those who had a PICC line inserted, 20 (5.6%) patients developed a catheter-related thrombosis, while in comparison only 4 (1.7%) patients developed this following LTSTC insertion, which was a statistically significant difference (p = 0.003) (Figure 1).

The median follow-up time for PICC patients was 53 days (95% CI: 44-62 days) with an initial 30-day incidence of DVT of 5% (of total number of PICC patients, n = 346), which subsequently increased to 14% at 180 days. Thus the number of patients presenting with catheter-related thrombosis increased the longer their PICC line was kept in.

Similar results were seen in LTSTC patients though the incidence rates were lower, where over a median follow-up period of 97 days (95% CI: 78-116 days) the 30-day incidence was only 1% (of total number of LTSTC patients, n = 237). This subsequently increased to 6% during the review period of 27 months, thus highlighting that the cumulative risk of thrombosis in this group did increase with time but at a slower rate.

### Risk factors associated with line-related thrombosis

In Table 2 we compared the prevalence of risk factors in patients who developed either a PICC or LTSTC related thrombosis. These factors were based on previous studies which examined thrombotic events in patients with malignancy [3,19]. There was no difference in the number of patients who were receiving Immunomodulatory Drugs (IMiDs) or anti-coagulant prophylaxis prior to developing a thrombosis. However there was a notable difference between the two groups with respect to prior history of venous thromboembolism (VTE), with this risk factor being more prevalent in the PICC compared to LTSTC group. Overall, however, these risk factors were found not to be statistically significant (p = 0.121).

**Table 2:** **Prevalence of risk factors in patients who developed a line-related thrombosis**

**Risk factor**	**Number of patients in PICC group (n = 20)**	**Number of patients in LTSTC group (n = 4)**
**Known background of inherited prothrombotic conditions (%)**	0 (0)	0 (0)
**History of VTE or PE (%)**	2 (10)	0 (0)
**Chemotherapy administered (%):**		
**Immunomodulatory drug**	2 (10)	2 (10)
**Hormonal therapy**	0 (0)	0 (0)
**Anti-platelet therapy (%)**	0 (0)	0 (0)
**Anti-coagulant prophylaxis (LMWH) (%)**	2 (10)	2 (10)

NB: PICC = Peripherally-Inserted Central Venous Catheter; LTSTC = Long Term Skin Tunneled Catheter; VTE = Venous Thromboembolism; PE = Pulmonary Embolism; LMWH = Low-Molecular Weight Heparin.

Other risk factors to consider include the type of underlying hematological malignancy. As shown in Table 1 there were a higher number of AML patients in the PICC group in comparison to the LTSTC group, while this was vice versa for patients with Multiple Myeloma, and this may potentially influence thrombosis risk. However, given the small number of outcomes, further statistical analysis to confirm whether this factor had an impact on thrombosis risk could not be performed.

Another risk factor is the stage of disease. Previously, it has been noted that patients with newly diagnosed disease are often at greatest risk of thrombosis, particularly within the first 3 months [3]. We examined the rates of catheter-related thrombosis between the following groups: Newly Diagnosed, Relapse and Remission. The rate of thrombosis between these groups were 5.3% vs 6.1% vs 6.5% respectively, which was not statistically significant (p = 0.958), suggesting that this did not influence thrombosis risk.

### Secondary outcomes

None of the LTSTC or PICC patients who had a catheter-related thrombosis developed a subsequent pulmonary embolism (PE). There were 9 (45%) line-related infections in the PICC group compared to 1 (20%) in the LTSTC group. Furthermore, 16 (80%) of the PICC compared to 2 (50%) of the LTSTC group had their line removed following a thrombotic event.

All patients who developed a catheter-related thrombosis were managed with 3-month therapeutic dose anticoagulation. At diagnosis, the median platelet count for these patients was 124.6×10^9^/L (range 15-320×10^9^/L) with all having a normal clotting range. There were no documented hemorrhagic complications associated with treatment, and none of our patients required thrombolysis or inferior vena cava (IVC) filter insertion.

Overall there was no statistically significant difference in the rates of post-thrombotic complications between the PICC and LTSTC groups (p = 0.597).

## Discussion

Over the years there has been an increasing use of PICCs due to the procedure being less invasive and the relatively low rate of line-related complications. Within our hospital trust, the current policy is to use PICCs first line, particularly for newly diagnosed patients who require urgent treatment, while LTSTCs are reserved for those undergoing stem cell rescue. However the risk of catheter-related thrombosis is often not considered. This is an important complication, especially in hemato-oncology patients, as it can potentially result in delayed administration of chemotherapy and expose them to the hazards of therapeutic anticoagulation [20].

When examining the current literature, there is minimal data available regarding the incidence of catheter-related thrombosis in hemato-oncology patients. There are studies which have been performed in critical care patients, with one case-control study reporting a thrombotic incidence of 2.8% in PICCs compared to 0% in LTSTCs [21]. Furthermore, a prospective randomized controlled trial in post-critical care patients who had routine surveillance for catheter-related thromboses also observed a higher incidence of thrombotic events in PICCs compared to short term CVCs (27.2% versus 9.6% respectively) [22].

Ours is the largest most recent study which has focused on comparing the incidence of catheter-related thromboses in hemato-oncology patients. The data we have produced is similar to previous studies, suggesting that PICCs confer an increased risk of catheter-related thrombosis compared to LTSTCs. Furthermore the majority of our patients in both groups presented within the first 100 days of insertion, with the cumulative incidence of thrombosis increasing over time. This would suggest an association between dwell time of line and risk of thromboembolic event, although interestingly the dwell time of line was significantly longer in the LTSTC group compared to the PICC group.

When considering the association between dwell time and risk of thrombotic event there has been mixed data, with some studies showing an association between prolonged dwell time and increased risk of thrombosis, while others have not. Overall the data produced so far does seem to favor a lack of an association between dwell time and risk of thrombosis [12,22,23]. However, within our own study, we were unable to exclude the possibility that longer dwell time contributed towards increased thrombotic risk as we were unable to perform an adjusted analysis.

There has been considerable interest in identifying other potential risk factors for thromboembolism, particularly in cancer patients, in order to prevent thrombotic events. These have been sub-divided into three categories: Patient-related, Disease-related and Treatment-related [3,19]. Given that we were examining hemato-oncology patients, we particularly focused on patient- and treatment-related risk factors, which included known inherited prothrombotic states, prior history of venous thromboembolic event and use of immunomodulatory drugs. Though it was unclear whether these risk factors would have the same impact in patients with central venous catheters, we assumed that these factors would contribute towards thromboembolic risk and therefore documented the incidence of each of these risk factors in both the PICC and LTSTC group. The use of IMiDs and anti-coagulant prophylaxis occurred with equal frequency in both groups, with none of our patients having a known inherited prothrombotic condition. However history of venous thromboembolism occurred more frequently in the PICC group compared to the LTSTC group, suggesting this is a contributing factor, despite it not being statistically significant.

Another point to consider in hemato-oncology patients is not only the risk of developing a catheter-related thrombosis, but the potential complications which can follow this. Our secondary outcomes examined the incidence of line-related infection, line removal, major hemorrhage and pulmonary embolism in hemato-oncology patients following development of a catheter-related thrombosis.

The risk of line-related infection has been previously shown to be significantly increased in those with a thrombosis, with colonization and sepsis rates reportedly doubling [24–26]. This would be further enhanced in hemato-oncology patients as they frequently have severe and long-lasting neutropenia, placing them at greater risk of infective complications generally. Our data reflected this with 9 patients in the PICC group compared to only 1 in the LTSTC group developing line-related infection following a thrombotic event, with all of these patients subsequently having their line removed.

Given that our unit performs a large number of stem cell rescues (over 200 per year), we also examined whether development of a thrombosis had an impact on the patient’s treatment plan. Of those who developed a catheter-related thrombosis, 4 patients from the PICC group had their treatment delayed, which included 3 stem cell rescues. The median duration of treatment delay was 13.8 days (range 4-20 days), which ultimately will have resulted in prolonged hospitalization, although this was not formally analyzed within our study. Hence this highlighted not only the short-term impact of a catheter-related thrombosis, but the potential long-term consequences also with delays to treatment. Therefore, this is another factor which needs to be taken into consideration when determining the type of venous access for a hemato-oncology patient.

The high rate of thrombosis in cancer patients with central venous access has led to several studies examining the potential use of systemic anticoagulant therapy [2]. Warfarin had previously been shown to successfully reduce the risk of catheter-related thrombosis in patients with solid tumors [27]. However, this can be a risky strategy in hematological patients, due to the intermittent and prolonged periods of chemotherapy-induced thrombocytopenia.

Abdelkefi et al. were able to overcome this in a well-controlled hospital setting where hemato-oncology patients were randomized to receive either intravenous unfractionated heparin or normal saline infusions daily, with a significantly lower incidence of catheter-related thrombosis reported in the former compared to the latter group (1.5% versus 12.6% respectively) [28]. Furthermore, there were no reported significant bleeding events, suggesting that low-dose unfractionated heparin could be safely administered in this group of patients. Our own data supports the safe use of therapeutic anticoagulation as we reported no major bleeding events, despite our patients being thrombocytopenic.

More recent studies, however, have shown no benefit from thromboprophylaxis in preventing catheter thrombosis [29–31]. All of these were double blind trials, hence providing higher quality evidence compared to the earlier published randomized trials which were not blinded. Therefore, given these conflicting results, the current guidelines released by the American College of Chest Physicians (ACCP) do not recommend the routine use of anticoagulation in cancer patients with central venous lines until additional randomized controlled trials can confirm that the benefits of anticoagulation outweigh the risks [32].

Given that our study was retrospective, the greatest limitation was the fact that we only identified symptomatic patients who underwent ultrasonographic examination. Recent studies which used prospective designs and screened for venous thrombosis in the absence of clinical symptoms suggest that catheter-related thrombosis is a complication that may be more prevalent than clinically perceived, with incidence rates as high as 64.5% [18,33].

In conclusion we observed a higher incidence of thrombotic events in hemato-oncology patients with PICCs compared to CVCs, which was associated with a higher rate of post-thrombotic complications, notably infection. Our findings have potential clinical implications where physicians have to weigh the risk and benefit of either PICC or CVC for central venous access, as this can potentially impact the long-term management of patients with hematological malignancies.

Future randomized controlled trials comparing PICCs against LTSTCs are required which are adequately powered to detect differences in both short-term and long-term outcomes (i.e. PE, hospitalization rates and mortality rates). Further data is also required to address the ongoing debate on the use of pharmacological thromboprophylaxis in patients with central venous access, with a particular focus on those with hematological malignancies.

## Abbreviations

ACCP: American college of chest physicians
AML: Acute myeloid leukemia
CRT: Catheter-related thrombosis
CVC: Central venous catheter
DVT: Deep vein thrombosis
IMiD: Immunomodulatory drug
IVC: Inferior vena cava
LTSTC: Long term skin tunneled catheter
PE: Pulmonary embolism
PICC: Peripherally-inserted central venous catheter
